# Reply to Hodac, N.; Wittekind, A. Comment on “Moz-Christofoletti, M.A.; Wollgast, J. Sugars, Salt, Saturated Fat and Fibre Purchased through Packaged Food and Soft Drinks in Europe 2015–2018: Are We Making Progress? *Nutrients* 2021, *13*, 2416”

**DOI:** 10.3390/nu14051117

**Published:** 2022-03-07

**Authors:** Maria Alice Moz-Christofoletti, Jan Wollgast

**Affiliations:** Joint Research Center, European Commission, 21027 Ispra, Italy; maria.moz-christofoletti@ec.europa.eu

We acknowledge the points raised by Hodac and Wittekind [1] and would like to address the concerns raised by the authors.

We thank the authors for the opportunity to discuss our findings in more detail. Figure 1 presents the 2015–2018 estimated changes in average sugars (g/100 mL), sales-weighted mean sugars (g/100 mL) and total sugars sold to consumers through retailers (g/per capita/day) when removing (i) 100% juice products (light blue bar) and (ii) 100% juices and concentrates (dark blue bar). It shows that the inclusion of 100% juice and concentrates (liquids/powders) within the soft drinks category as in Moz-Christofoletti and Wollgast [2]—driven by the categorisation used by Euromonitor—has not severely diluted the relative progress estimated for this product group. Compared to our published estimates (white bar), a dilution effect of approximately 2.3 percentage points (p.p.) is observed for average sugars, suggesting that products that can be reformulated have decreased their sugar content. However, (i) the sales-weighted mean sugars estimates are considerably less affected (0.4 p.p.) and (ii) the per capita sales of total sugars have not changed after excluding 100% juices and concentrates. This is particularly important since these two metrics are used to assess progress against the ambition for sugar reduction from a public health perspective [3,4]. As in [2], the results from Figure 1 confirm that greater sugar reduction is observed in drinks with relatively lower market volume, while products with a higher market share have reduced their sugar content to a lesser extent.

A more in-depth comparison between our estimates and the one provided by [1] is limited by the total absence of information on methodology and data sources used. Beyond methodological aspects, possible discrepancies might arise from different nutrient coverage (total vs. added sugars), distribution channels considered (only retail vs. retail and food service), country and category coverages, as well as timeframe. More transparency on data and methodology would greatly help researchers, consumers, stakeholders and the public at large to obtain more reliable and precise information about the industry’s reformulation efforts and its achievements and credibility.

## Figures and Tables

**Figure 1:**
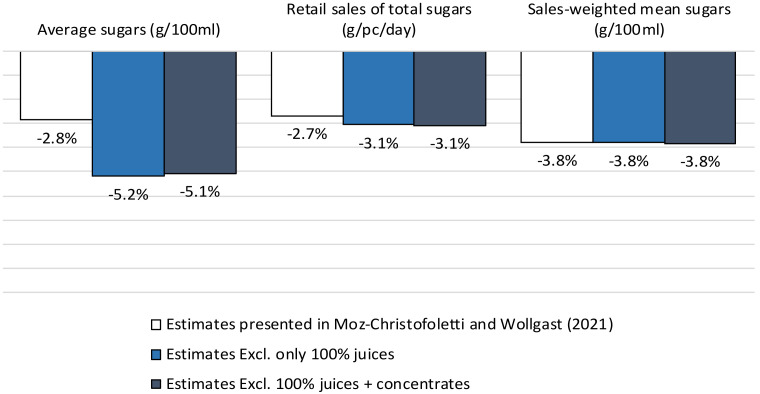
Evolution of the average sugars (g/100 mL), retail sales of total sugars (g/pc/day) and sales-weighted mean sugars (g/100 mL) in different categories of soft drinks between 2015 and 2018 in 22 European countries. Source: Elaborated by the authors based on data from [5]. Note: The values were scaled by the market coverage to represent 100% of the retail market. (1) As defined by Euromonitor, the category Soft Drinks covers Asian Speciality Drinks, Carbonated RTD Tea, Coconut and Other Plant Waters, Flavoured Bottled Water, Functional Bottled Water, Ginger Ale, Juice Drinks (up to 24% Juice), Lemonade/Lime, Liquid Concentrates, Low Calorie Cola Carbonates, Nectars, Not from Concentrate 100% Juice, Orange Carbonates, Other Non-Cola Carbonates, RTD Coffee, Reconstituted 100% Juice, Regular Cola Carbonates, Regular Energy Drinks, Regular Sports Drinks, Still RTD Tea and Tonic Water/Other Bitters. (2) EU19 plus Norway, Switzerland and the United Kingdom.

## Data Availability

The data that support the findings of this study are available from Euromonitor International (https://www.euromonitor.com/, accessed on 20 May 2020), but restrictions apply to the availability of these data, which were used under license for the current study, and so are not publicly available. However, data are available from the authors upon reasonable request and with permission from Euromonitor International.
